# Update on Vaccine-Derived Poliovirus Outbreaks — Democratic Republic of the Congo and Horn of Africa, 2017–2018

**DOI:** 10.15585/mmwr.mm6809a2

**Published:** 2019-03-08

**Authors:** Chukwuma Mbaeyi, Mary M. Alleman, Derek Ehrhardt, Eric Wiesen, Cara C. Burns, Hongmei Liu, Raimi Ewetola, Lerato Seakamela, Rennatus Mdodo, Modjirom Ndoutabe, Pierre Kandolo Wenye, Yogolelo Riziki, Peter Borus, Christopher Kamugisha, Steven G. F. Wassilak

**Affiliations:** ^1^Global Immunization Division, Center for Global Health, CDC; ^2^Division of Viral Diseases, National Center for Immunization and Respiratory Diseases, CDC; ^3^CDC-Democratic Republic of the Congo, Kinshasa; ^4^National Institute for Communicable Diseases, Johannesburg, South Africa;^5^Liaison Office for Somalia, World Health Organization, Nairobi, Kenya; ^6^Global Polio Eradication Initiative Coordination Office, World Health Organization, Kinshasa, Democratic Republic of the Congo; ^7^Emergency Operations Center for Polio, Ministry of Health, Kinshasa, Democratic Republic of the Congo; ^8^Institut National de Recherche Biomédicale, Ministry of Public Health, Kinshasa, Democratic Republic of the Congo; ^9^Kenya Country Office, World Health Organization, Nairobi, Kenya; ^10^Horn of Africa Coordination Office, World Health Organization, Nairobi, Kenya.

Widespread use of live attenuated (Sabin) oral poliovirus vaccine (OPV) has resulted in marked progress toward global poliomyelitis eradication (*1*). However, in underimmunized populations, extensive person-to-person transmission of Sabin poliovirus can result in genetic reversion to neurovirulence and paralytic vaccine-derived poliovirus (VDPV) disease (*1*). This report updates (as of February 26, 2019) previous reports on circulating VDPV type 2 (cVDPV2) outbreaks during 2017–2018 in the Democratic Republic of the Congo (DRC) and in Somalia, which experienced a concurrent cVDPV type 3 (cVDPV3) outbreak* (*2*,*3*). In DRC, 42 cases have been reported in four cVDPV2 outbreaks; paralysis onset in the most recent case was October 7, 2018 (*2*). Challenges to interrupting transmission have included delays in outbreak-response supplementary immunization activities (SIAs) and difficulty reaching children in all areas. In Somalia, cVDPV2 and cVDPV3 were detected in sewage before the detection of paralytic cases (*3*). Twelve type 2 and type 3 cVDPV cases have been confirmed; the most recent paralysis onset dates were September 2 (cVDPV2) and September 7, 2018 (cVDPV3). The primary challenge to interrupting transmission is the residence of >300,000 children in areas that are inaccessible for vaccination activities. For both countries, longer periods of surveillance are needed before interruption of cVDPV transmission can be inferred.

## Vaccine-Derived Polioviruses

VDPV types 1 or 3 are polioviruses that are >1% divergent (≥10 nucleotide differences in the genetic sequence) from the corresponding Sabin OPV strain in the viral protein 1 (VP1) genomic coding region (*1*,*4*). VDPV2s are >0.6% divergent (≥6 nucleotide differences in the VP1 coding region) (*1*,*4*). When polioviruses replicate during transmission, nucleotide substitutions in the viral genome accumulate at approximately 1.1% (10 nucleotides of the VP1 coding region) per year, which can provide the means to determine how long a strain has been circulating. VDPVs are classified as circulating (cVDPVs) when community transmission is demonstrated by genetic linkages of VDPVs isolated from paralytic cases, community contacts, or environmental (sewage) samples (*4*).

## 2016 Global Switch from Trivalent OPV to Bivalent OPV

The type 2 component of trivalent OPV (tOPV) (containing vaccine virus types 1, 2, and 3) was responsible for >90% of cVDPV cases occurring during 2006–2015 (*5*–*7*). After the declaration of eradication of wild poliovirus type 2 in 2015 (*6*,*7*), a globally synchronized switch from tOPV to bivalent OPV (bOPV) (containing types 1 and 3) occurred in all OPV-using countries by May 1, 2016 (*6*,*7*). A single dose of inactivated poliovirus vaccine (IPV), which includes all three poliovirus serotypes, was introduced into routine immunization schedules in OPV-using countries to mitigate the risk for a gap in immunity to poliovirus type 2 (*6*). Children who seroconvert after IPV administration are protected from paralytic disease but still can contribute to the transmission of poliovirus. Monovalent type 2 OPV (mOPV2) is held in a global stockpile for implementation of outbreak response SIAs for poliovirus type 2 outbreaks after the switch (*8*).

## cVDPV2 Outbreaks in the Democratic Republic of the Congo

**FIGURE 1:**
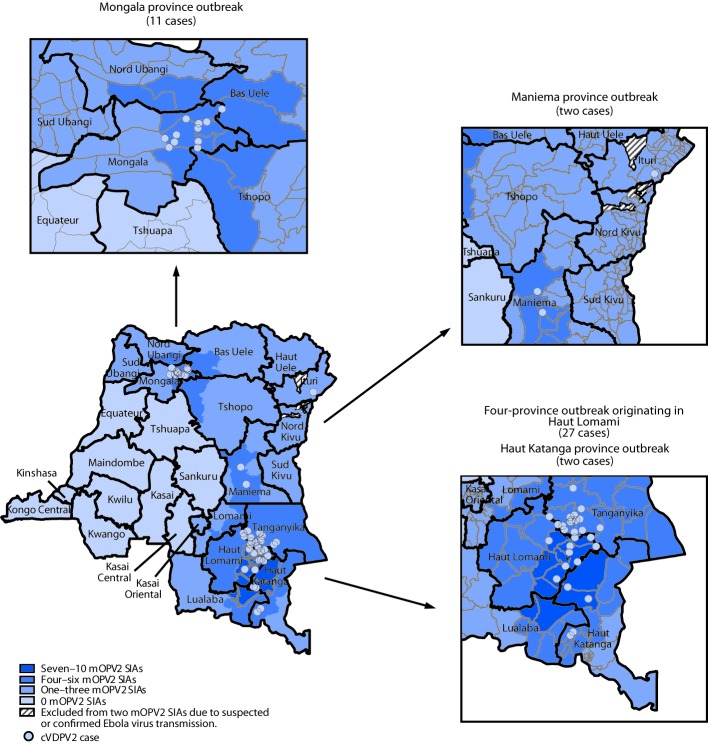
Circulating vaccine-derived poliovirus type 2 (cVDPV2) cases, by location and number of response supplementary immunization activities (SIAs) with monovalent oral poliovirus vaccine type 2 (mOPV2) — Democratic Republic of Congo, 2017–2018* * Each dot represents one confirmed paralytic cVDPV2 case. Dots are randomly positioned within health zones and do not represent exact locations where cases occurred.

**Maniema province outbreak (two cases):** The first patient in this outbreak had paralysis onset on March 26, 2017, and the second had paralysis onset on April 18, 2017 (*2*). Genetic analyses of the cVDPV2 isolates identified a 7-nucleotide difference from the Sabin type 2 strain, suggesting recent emergence. After the onset of the most recent case, four to five mOPV2 supplementary immunization activities (SIAs) were conducted in the health zones (subprovince areas) nearest to the identified cases and two in the remainder of the province (Figure 1).

**Four-province outbreak originating in Haut Lomami (27 cases):** The first patient had paralysis onset on February 20, 2017, in Haut Lomami province; the VDPV2 isolate from this case had a 15-nucleotide difference from Sabin 2, indicating >1 year of undetected circulation. Subsequent to this case, 26 additional cases with genetically linked cVDPV2 isolates were identified, with paralysis onset from March 8, 2017, to May 27, 2018, in Haut Lomami province (eight cases), in two adjacent provinces (Haut Katanga [two] and Tanganyika [15]), and in Ituri province in northeastern DRC (one). In response to these cases, up to 10 mOPV2 SIAs were conducted in the outbreak area; three mOPV2 SIAs were conducted in the broader outbreak area after the onset of the most recent case (Figure 1) (*2*). The isolate from the Ituri patient was genetically linked to the Haut Lomami outbreak area; however, no epidemiologic link was established. Up to three mOPV2 SIAs were conducted after the onset of the single case in Ituri province, except in health zones where Ebola virus transmission had been confirmed or suspected in 2018 (*9*).

**Mongala province outbreak (11 cases):** The first case of paralysis onset associated with this outbreak occurred on April 26, 2018, and the patient’s VDPV2 isolate had a 19-nucleotide difference from Sabin 2, indicating nearly 2 years of undetected circulation. Ten additional cases with genetically linked viruses were reported, with paralysis onset during June 14–September 13, 2018. Four mOPV2 SIAs were conducted in health zones with identified cases and two to four in the remainder of Mongala and neighboring provinces; two mOPV2 SIAs have been conducted in the entirety of the outbreak area after the onset of the most recent case (Figure 1).

**Haut Katanga province outbreak (two cases):** In this outbreak, the first patient had paralysis onset on October 6, 2018, and the second on October 7. The VDPV2 isolates had 7- and 8-nucleotide differences from the Sabin 2 strain, indicating emergence in 2018 after use of mOPV2 for SIAs in response to the Haut Lomami area outbreak, with suboptimal coverage achieved. Two SIAs were conducted after the onset of these cases (Figure 1).

## cVDPV2 and cVDPV3 Outbreaks in the Horn of Africa

**FIGURE 2:**
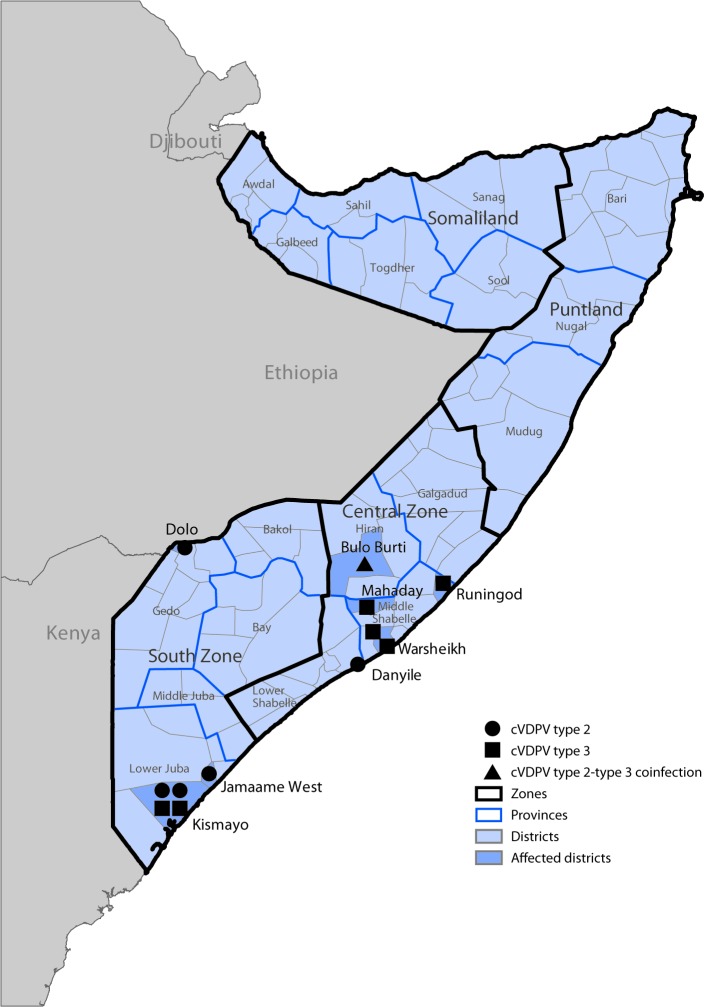
Circulating vaccine-derived poliovirus (cVDPV) type 2 and type 3 cases, as of February 26, 2019, by location — Somalia, 2018* * Each symbol represents one confirmed paralytic cVDPV case. Symbols are randomly positioned within districts and do not represent exact locations where cases occurred.

**FIGURE 3:**
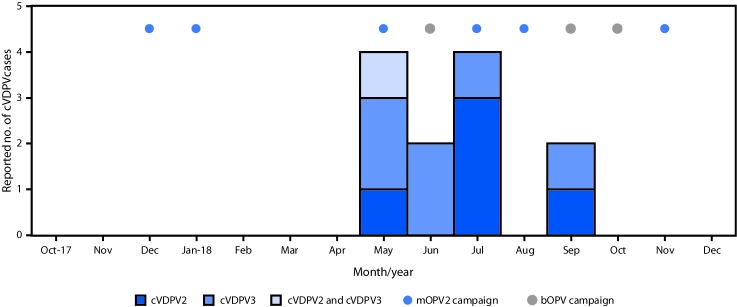
Circulating vaccine-derived poliovirus (cVDPV) cases and outbreak response supplementary immunization activities, by month — Somalia, 2017–2018 **Abbreviations:** bOPV = bivalent oral poliovirus vaccine, types 1 and 3; cVDPV2 = circulating vaccine derived poliovirus type 2; cVDPV3 = circulating vaccine derived poliovirus type 3; mOPV2 = monovalent oral poliovirus vaccine type 2.

Environmental surveillance, the testing of sewage samples for polioviruses, detected genetically linked cVDPV2 in samples taken from two different environmental surveillance sites in Banadir province, Somalia, in October 2017 and January 2018 and genetically linked cVDPV3 from two different sites in April 2018. Genetic analyses of the viruses indicated undetected circulation of cVDPV2 for >3 years (36–44-nucleotide differences from Sabin 2) and of cVDPV3 for >1 year (15–17-nucleotide differences from Sabin 3) (*3*). No genetically linked paralytic cVDPV cases were detected until a coinfection with cVDPV2 and cVDPV3 was identified in a patient from the central province of Hiran, with paralysis onset on May 11, 2018 (Figure 2) (*3*). As of January 31, 2019, a total of 12 cVDPV cases had been identified in Somalia: five cVDPV2 cases, six cVDPV3 cases, and the cVDPV2/cVDPV3 coinfection (Figure 2) (Figure 3). The most recent paralysis onsets occurred on September 2 (cVDPV2) and September 7, 2018 (cVDPV3). Three patients resided in districts that were inaccessible for polio vaccination for >5 years, and none had ever received OPV.

Twenty-one sewage samples from environmental surveillance sites in Banadir province tested positive for genetically linked cVDPV2, the most recent collected on October 11, 2018. One sewage sample collected in Kamakunji district, Kenya, in March 2018 tested positive for cVDPV2 genetically linked to strains circulating in Somalia (*3*); however, no cVDPV2 cases were detected in Kenya. Genetically linked cVDPV3 isolates were identified in 12 sewage samples from Banadir province, the most recent collected on August 23, 2018. No cVDPV3 isolates have been detected by environmental or acute flaccid paralysis (AFP) surveillance in Kenya, and neither cVDPV2 nor cVDPV3 has been detected in Ethiopia.

In response to the Horn of Africa cVDPV2 outbreak, six mOPV2 outbreak response SIAs were conducted in Somalia during December 2017–November 2018, including two conducted after the most recent case onset. Two of these SIAs were synchronized with subnational mOPV2 outbreak response SIAs in Kenya and Ethiopia during July–September 2018. Before that, when cVDPV2 was identified by environmental surveillance in Kenya, a focal mOPV2 outbreak response SIA was conducted in Kamakunji district in May 2018.

After cVDPV3 detection in Somalia, three bOPV outbreak response SIAs were conducted there during April–October 2018, two of which were synchronized with subnational bOPV SIAs in Kenya during September–October 2018. Both SIAs were implemented after paralysis onset of the most recent cVDPV3 case in Somalia.

## Discussion

During 2005–2013, multiple cVDPV2 outbreaks occurred in DRC and Somalia (*2*,*10*). Because of chronically low childhood routine immunization coverage in both countries, preventive tOPV SIAs were implemented annually to boost immunity before the tOPV/bOPV switch in 2016 (*2*,*10*). The cVDPV outbreaks during 2017–2018 indicate that children residing in the outbreak-affected areas were not effectively reached with tOPV before the switch (and for type 3, with bOPV after the switch) through childhood routine immunization services or preventive SIAs. After the tOPV/bOPV switch, preventive SIAs using tOPV can no longer be implemented; although IPV can provide protection from paralytic disease to infected children who have received it, low routine immunization coverage precluded IPV serving as a substantive means of preventing cVDPV cases in both countries. In addition to DRC and Somalia, cVDPV2 outbreaks also were identified during 2017–2018 in Mozambique, Niger, Nigeria, and Syria. Although improving delivery of bOPV through routine immunization services would prevent cVDPV1 or cVDPV3 outbreaks, this would require considerable time, effort, and resources. Preventive bOPV SIAs can raise population immunity more quickly in countries and areas with low routine immunization coverage.

cVDPV2 transmission in the DRC outbreaks might have ceased; however, a longer period of surveillance is needed before interruption of transmission can be inferred. Because of serious limitations in mOPV2 SIA quality (i.e., low population coverage), delays in SIA implementation, and a smaller geographic scope than that needed for some SIAs, many more SIAs were needed to achieve apparent interruption of transmission than are usually required. As well, when SIA coverage in the target population is low, there is a risk that the mOPV2 response SIAs themselves will seed new cVDPV2 outbreaks; in DRC, the Haut Katanga outbreak resulted from suboptimal outbreak response SIAs for the Haut Lomami area outbreak.

In Somalia, AFP surveillance performance indicators have been met, even in insecure areas where community-based surveillance is conducted. However, undetected cVDPV2 and cVDPV3 transmission for approximately 1–3 years indicates high likelihood that the emergence and circulation of VDPVs occurred among unimmunized children residing in inaccessible areas. To extend the reach of the outbreak response as much as possible, outbreak response SIAs included vaccination of children living in inaccessible areas when they were at transit points (e.g., bus stations) and at markets, and rapid response vaccination in a few areas where children were not usually accessible for vaccination. However, >300,000 unimmunized children are estimated to reside in these areas. An extended period of AFP surveillance and environmental surveillance will be needed to indicate that cVDPV transmission has been interrupted in Somalia.

In both countries, if additional response is required, programs need to ensure the quality and reach of timely SIAs. The continued use of aggressive strategies, such as transit-point vaccination, to reach underimmunized populations, should be considered.

**Summary**

**What is already known about this topic?**

Prolonged person-to-person transmission of polio vaccine viruses in underimmunized populations can lead to emergence of outbreaks of paralysis from circulating vaccine-derived poliovirus (cVDPV).

**What is added by this report?**

During 2017–2018, four cVDPV type 2 outbreaks, with 42 cases to date, occurred in six provinces of the Democratic Republic of the Congo and required multiple response supplementary immunization activities (SIAs). In Somalia, concurrent cVDPV type 2 and cVDPV type 3 outbreaks occurred, first identified by sewage testing months before occurrence of 12 paralytic cases to date.

**What are the implications for public health practice?**

To promptly interrupt cVDPV transmission, country programs must effectively plan and implement timely response SIAs to optimize their quality and reach.

## Footnotes

* Because of the outbreak in DRC, on July 19, 2017, CDC issued a Level 2 Travel Health Notice recommending that all travelers to the DRC be fully vaccinated against polio. A similar notice was issued for Somalia on July 23, 2018. Before traveling to the DRC or Somalia, adults who completed their routine polio vaccine series as children are advised to receive a single, lifetime adult booster of polio vaccine.
